# QuantumXCT: Learning Interaction-Induced State Transformation in Cell-Cell Communication via Quantum Entanglement and Generative Modeling

**Published:** 2026-04-09

**Authors:** Selim Romero, Shreyan Gupta, Robert S. Chapkin, James J. Cai

**Affiliations:** 1 Department of Veterinary Integrative Biosciences, Texas A&M University; 2 Department of Nutrition, Texas A&M University; 3 CPRIT Single Cell Data Science Core, Texas A&M University; 4 Department of Electrical and Computer Engineering, Texas A&M University

**Keywords:** Quantum Computing, Quantum Machine Learning, Bioinformatics, Single-cell

## Abstract

Inferring cell–cell communication (CCC) from single-cell transcriptomics remains fundamentally limited by reliance on curated ligand–receptor databases, which primarily capture co-expression rather than the system-level effects of signaling on cellular states. Here, we introduce QuantumXCT, a hybrid quantum–classical generative framework that reframes CCC as a problem of learning interaction-induced state transformations between cellular state distributions. By encoding transcriptomic profiles into a high-dimensional Hilbert space, QuantumXCT trains parameterized quantum circuits to learn a unitary transformation that maps a baseline non-interacting cellular state to an interacting state. This approach enables the discovery of communication-driven changes in cellular state distributions without requiring prior biological assumptions. We validate QuantumXCT using both synthetic data with known ground-truth interactions and single-cell RNA-seq data from ovarian cancer–fibroblast co-culture model. The QuantumXCT model accurately recovered complex regulatory dependencies, including feedback structures, and identified dominant communication hubs such as the PDGFB-PDGFRB-STAT3 axis. Importantly, the learned quantum circuit is interpretable: its entangling topology was translated into biologically meaningful interaction networks, while post hoc contribution analysis quantified the relative influence of individual interactions on the observed state transitions. Notably, by shifting CCC inference from static interaction lookup to learning data-driven state transformations, QuantumXCT provides a generative framework for modeling intercellular communication. This work establishes a new paradigm for *de novo* discovery of communication programs in complex biological systems and highlights the potential of quantum machine learning in the context of single-cell biology.

## 1 Introduction

Inferring cell-cell communication (CCC) from single-cell transcriptomic data is challenging but crucial for understanding complex biological systems. Over the past decade, a growing ecosystem of computational tools has emerged to address this problem, ranging from ligand-receptor scoring methods such as CellChat [1, 2] and CellPhoneDB [3] to multi-method frameworks such as LIANA [4] and semi-supervised approaches such as scTenifoldXct [5]. Despite this diversity, most current methods remain tethered to curated databases of known ligand-receptor pairs [6]. This reliance creates a critical bottleneck: it constrains discovery to existing knowledge and limits the identification of novel or context-specific signaling pathways. A systematic comparison of sixteen inference resources and seven methods showed that both resource and method selection strongly influence predicted interactions, underscoring the instability of database-dependent approaches [4]. Moreover, as we have recently demonstrated in a simulation study [7], standard CCC inference methods fail to recover programmed causal pathways and instead report spurious associations driven by expression statistics alone. At their core, existing methods are primarily designed to test co-expression of known interacting genes rather than learn the underlying principles of communication directly from the data [6].

To overcome these limitations, we propose a paradigm shift in CCC inference. Rather than searching for interacting ligand-receptor pairs, we treat CCC inference as a problem modeling the emergent transformation of cellular states induced by interaction. Specifically, we hypothesize that the communication signal is quantitatively captured by the difference between two transcriptomic probability distributions: a baseline state of cells in isolation (e.g., mono-culture) and a perturbed state measured in an interacting context (e.g., co-culture). Defining this high-dimensional distributional shift—without imposing prior biological assumptions—requires a new class of computational modeling.

While classically intractable, this task is well suited to the strengths of quantum computing. Quantum systems are inherently probabilistic and excel at representing the complex, high-dimensional probability landscapes that are difficult to capture with classical computers. A quantum processor with *n* qubits can naturally explore a state space of 2^*n*^ dimensions, mirroring the possibly immense combinatorial complexity of a cell’s transcriptome. Leveraging superposition and entanglement, a parameterized quantum circuit (PQC) [8] can be trained to approximate intricate probability distributions, making it a promising tool for capturing subtle, system-wide shifts induced by cell-cell interactions. This capability is harnessed within a hybrid quantum-classical optimization loop [9], in which a classical optimizer iteratively refines the quantum circuit’s parameters to minimize a data-driven cost function.

Crucially, the trained PQC is not a black box; it provides an interpretable model of the underlying dynamics. The optimized parameters of the learned unitary transformation encapsulate the rules governing cellular state changes, reflecting both intercellular signaling and downstream intracellular responses, such as gene regulatory network (GRN) rewiring. By learning the transformation itself, our quantum machine learning (QML) approach yields a data-driven representation of interactions. Moreover, by comparing circuits optimized under different biological conditions, we can systematically identify how communication programs are altered across development or disease.

**Fig. 1:**
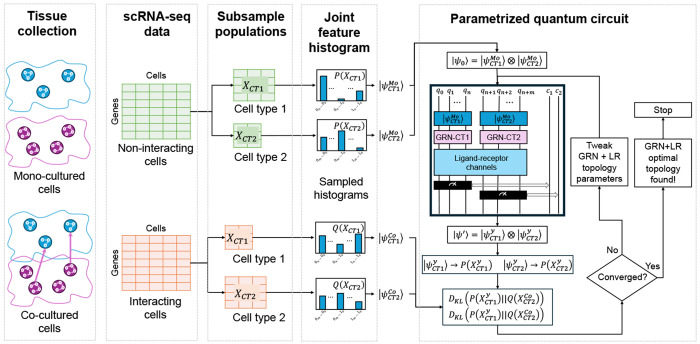
A Quantum Generative Framework for Modeling Cell-Cell Communication. Our workflow reframes CCC as a learned transformation between cellular states. We derive two sets of empirical probability distributions from single-cell RNA sequencing: a **baseline distribution**, *P* (*X*^Mo^), from non-interacting (mono-culture) cells, and a **target distribution**, *Q*(*X*^Co^), from interacting (co-culture) cells. A parameterized quantum circuit (PQC) is then trained to model this transformation. The circuit is initialized to the global baseline state |Ψ_Mo_⟩ (derived from *P* (*X*^Mo^)) and applies a parameterized unitary operator, *U*(*τ, θ*), to produce a final state whose simulated marginal distributions are denoted *P*ψ′. In a hybrid quantum-classical loop, an optimizer iteratively adjusts the circuit’s topology (*τ*) and parameters (*θ*) to minimize the Kullback-Leibler (KL) divergence, *D*_KL_ (*P*ψ′ ∥ *Q*Co), between the circuit’s simulated output and the empirical target. Upon convergence, the optimized unitary, *U* (*τ*_opt_, *θ*_opt_), provides a quantitative, data-driven model of the communication dynamics.

To achieve this, we introduce a novel, database-free hybrid quantum-classical framework for learning CCC dynamics. Our approach centers on training a PQC to function as a *generative transformation model*. Rather than adopting a standard classification-oriented Quantum Neural Network (QNN) architecture, we pioneer a method where the circuit learns to execute the unitary transformation that evolves a non-interacting cellular state into an interacting one. The optimization of the circuit’s entangling topology and its gate parameters is guided by a custom-designed cost function based on the Kullback-Leibler (KL) divergence, which minimizes the difference between the circuit’s output distribution and the empirically observed interacting cells’ data (Fig. 1). We demonstrate this methodology on single-cell RNA sequencing (scRNA-seq) data, establishing a new avenue for the *de novo* discovery of cellular communication channels.

## 2 Methods

Our methodology infers CCC by learning a quantum-mechanical transformation that models how cellular states shift from a non-interacting to an interacting environment. The framework comprises three main stages: (1) pre-processing single-cell transcriptomic data to define baseline and target distributions; (2) specify a PQC to represent the state transformation; and (3) use a multi-stage hybrid optimization strategy to learn circuit parameters that best capture the communication dynamics.

### 2.1 Data Processing and Quantum State Encoding

Our framework takes as input scRNA-seq data from two distinct cell types (e.g., CT1 and CT2) collected under, both non-interacting (e.g., mono-culture) and interacting (e.g., co-culture) conditions (Fig. 1). The data were pre-processed using Scanpy [10], including total-count normalization and logarithmic transformation, *X′* = log(1+*X*). After pre-processing, we selected, for each cell type, a subset of genes/features most relevant to the biological context under study.

To map the continuous expression profile onto the discrete basis states of the quantum system, we binarized the expression of each selected gene. Each gene is considered “active” (state 1) or “inactive” (state 0) using a defined threshold; here, a gene was considered active if its log-normalized expression was greater than zero. This binarization was biologically principled since scRNA-seq data is inherently sparse, and zero observations often reflect genuinely low-expression states rather than purely technical dropout [11]. Binary representations of scRNA-seq data have been shown to faithfully capture cell-type-level biological variation [12], and the active/inactive threshold typically mirrors the characteristic ON/OFF expression switching observed in regulatory genes at single-cell resolution [13]. This procedure converts each cell’s transcriptomic profile into a bitstring, *s* ∈ {0, 1}^*d*^, where *d* is the number of selected genes. From the binarized data, we subsequently computed empirical joint frequency counts, *C*(*s*) for each unique cellular state *s*.

These frequency counts are then used to construct the two primary inputs for our quantum model: the initial quantum state and the classical target distributions.

1. **Baseline State Amplitudes:** To prepare the initial state of the quantum circuit, counts from the non-interacting (mono-culture) cells, *C*_Mo_(*s*), were L2-normalized to define the state vector’s amplitudes, $\alpha_{s}^{\mathrm{Mo}}$:
(1) $$\alpha_{s}^{\mathrm{Mo}}=\frac{C_{\mathrm{Mo}}(s)}{\sqrt{\sum_{s^{\prime }\in \{0,1{\}}^{d}}C_{\mathrm{Mo}}{\left(s^{\prime }\right)}^{2}}}.$$
  These amplitudes define the individual baseline state vectors, $\left|\psi_{\mathrm{CT}1}^{\mathrm{Mo}}\right\rangle$ and $\left|\psi_{\mathrm{CT}2}^{\mathrm{Mo}}\right\rangle$.
2. **Target Probability Distributions:** To define the classical target for our cost function, the counts from the interacting (co-culture) cells, *C*_Co_(*s*), were first L2-normalized to produce a vector of target amplitudes, $\alpha_{s}^{\mathrm{Co}}$. These amplitudes were then squared to yield the final target probability distributions, *Q*_Co_(*s*):
(2) $$\alpha_{s}^{\mathrm{Co}}=\frac{C_{\mathrm{Co}}(s)}{\sqrt{\sum_{s^{\prime }}C_{\mathrm{Co}}{\left(s^{\prime }\right)}^{2}}}\;\text{and}\;Q_{\mathrm{Co}}(s)={\left|\alpha_{s}^{\mathrm{Co}}\right|}^{2}.$$

### 2.2 The Quantum Transformation Model

Our model is a PQC [8] acting on a combined system of *N + M* qubits, comprising an *N*-qubit logical register for CT1 and an *M*-qubit logical register for CT2. The circuit’s evolution is described by the unitary transformation:

(3) $$\left|\psi^{\prime }\right\rangle =U(\tau ,\theta )\left|\Psi_{\mathrm{Mo}}\right\rangle .$$

Here, $\left|\Psi_{\mathrm{Mo}}\right\rangle$ is the **global initial state**, representing the two cell types in isolation. It was constructed by taking the tensor product of the individual baseline state vectors (Eq. 1):

(4) $$\left|\Psi_{\mathrm{Mo}}\right\rangle =\left|\psi_{\mathrm{CT}1}^{\mathrm{Mo}}\right\rangle ⊗\left|\psi_{\mathrm{CT}2}^{\mathrm{Mo}}\right\rangle .$$

The operator U(τ,θ) models the intercellular communication and is composed of a sequence of entangling gates connecting the two logical registers. It is defined by its discrete **topology** *τ* and continuous **parameters** *θ*.

### 2.3 Hybrid Optimization Strategy

The PQC was trained using a classical optimization loop [9] to find the optimal circuit parameters ($\tau_{\text{opt}},\theta_{\text{opt}}$) that minimize a cost function based on the Kullback-Leibler (KL) divergence [14].

### 2.3.1 Objective Function

The cost function, ℒ(τ,θ), measures the dissimilarity between the marginal distributions of the circuit’s output state, $\left|\psi^{\prime }\right\rangle$, and the target marginal distributions from the interacting data (Eq. 2):

(5) $$ℒ(\tau ,\theta )=D_{\mathrm{KL}}\left(P_{\psi^{\prime }}(\mathrm{CT}1)\|Q_{\mathrm{Co}}(\mathrm{CT}1)\right)+D_{\mathrm{KL}}\left(P_{\psi^{\prime }}(\mathrm{CT}2)\|Q_{\mathrm{Co}}(\mathrm{CT}2)\right),$$

where $P_{\psi^{\prime }}\left({\mathrm{CT}}_{k}\right)$ is the marginal probability distribution for cell type *k* obtained by simulating the PQC. Note that the cost was computed over marginal distributions independently for each cell type. Cross-cell-type correlations were not directly measured but are instead implicitly encoded by the entangling topology *τ*, which must create the inter-register dependencies required to simultaneously satisfy both marginal constraints.

#### 2.3.2 Topology Search (*τ*)

Finding the optimal entangling topology *τ* is an NP-hard optimal sequence search problem [15], as it required selecting both the best subset of gates and their optimal application order. Because exhaustive brute-force searches are intractable, we adopted a two-stage strategy: heuristic pruning of the search space, followed by the application of one of several search algorithms.

For the discrete topology search, we used controlled-RX (CRX) gates with a fixed angle of *π*/2 as the basic building blocks. This choice enabled the detection of weaker correlations via partial entanglement, as detailed in the circuit construction pipeline in Appendix A.1.1.

#### Heuristic Pruning of Candidate Gates

To guide the search, we first identified a high-potential candidate set of entangling gates, *C*, by analyzing the difference between the global density matrices of the non-interacting and interacting systems (see Appendix B for details). By applying a threshold to the elements of the difference matrix, Δ*ρ* (Eq. B4), we generated a reduced list of CNOT gates that were most likely to facilitate the required state transformation. This pruned set *C* was the input to all subsequent search algorithms.

#### Search Algorithms

We subsequently developed and compared three distinct strategies to search for the optimal topology within the pruned candidate set *C*:

1. **Algorithm 1: Iterative Local Search:** A classical greedy heuristic [16] that simultaneously explored gate selection and ordering by testing addition, insertion, and deletion moves in each iteration (see Appendix C for full details).
2. **Algorithm 2: Multi-Epoch Sequential Construction:** A faster, stochastic greedy search that used multiple random starting points to build candidate sequences, followed by a refinement pruning step applying an Occam’s Razor parsimony criterion [17] (see Appendix D for full details).
3. **Algorithm 3: QUBO-based Variational Selection:** A quantum-native approach [18, 19] that decoupled the problem into a gate selection stage, solved with VQE or QAOA, and a final classical permutation stage (see Appendix E for full details).

Table 1 summarizes the strengths and weaknesses of each algorithm. Based on our empirical results, we selected Algorithm 2 for the primary analyses because it offers a strong balance between computational efficiency and solution quality. In practice, all three algorithms achieved similar levels of KL minimization. Algorithm 1 was the most stable but also the most computationally intensive. Algorithm 2 was faster and provides a reliable estimate of the achievable minimization, and Algorithm 3 was a forward-looking approach that used quantum-assisted gate selection to narrow down permutation candidates, with the potential to outperform Algorithm 1 given a sufficiently large initial candidate pool.

#### 2.3.3 Parameter Optimization (*θ*)

For finer-grained modeling, the fixed-angle CRX(*π*/2) gates used in the discrete topology search are promoted to a continuous ansatz. This was achieved by replacing each fixed gate with a fully parameterized CRX(*θ_i_*) gate, allowing the model to learn the precise interaction strength. The final optimization step solved for the optimal vector of continuous parameters:

(6) $$\theta_{\mathrm{opt}}=\arg\;\underset{\theta }{\min}ℒ\left(\tau_{\mathrm{opt}},\theta \right),$$

where *θ* is the angle set to be optimized and initialized to zero (*θ_i_* = 0). This continuous optimization problem was solved using a classical gradient-free algorithm [20], specifically L-BFGS-B [21] or COBYLA [20], applied to the fixed topology $\tau_{\text{opt}}$.

### 2.4 Implementation Details

All data processing and analyses were performed in Python. Single-cell data were handled using scanpy [10]. The quantum circuits and simulations were implemented in IBM’s Qiskit framework [22, 23], using the qiskit_aer simulator [24]. Classical optimization used routines from SciPy.optimize [25]. Code for our quantum generative framework is available at GitHub (https://github.com/cailab-tamu/QuantumXCT).

## 3 Results

We evaluated the performance of QuantumXCT in two distinct stages. First, we used a controlled synthetic dataset as a proof-of-concept to show that the framework can learn predefined interaction dynamics and recover ground-truth rules (details in Appendix F). Second, we applied the model to real scRNA-seq datasets to assess its ability to identify novel CCC channels in complex biological environments.

### 3.1 Benchmarking via Synthetic State-to-State Transformations

To establish the utility of our hybrid quantum-classical approach, we first benchmarked its ability to learn a high-dimensional probability distribution transformation using simulated cellular data. This synthetic dataset (Appendix F) provided a ground-truth environment, in which gene-gene and cell-cell interactions were explicitly defined, enabling quantitative evaluation of the accuracy of our topology search algorithms.

#### 3.1.1 Data Encoding and Experimental Setup

Following the methodology in Section 2.1, we binarized the transcriptomic counts of selected gene sets into ordered bit-strings, where each bit represents the activity of a gene. These bit-strings define unique cellular states, which were then encoded as the basis states of a quantum system. The normalized frequency of these states determined the amplitudes of a state vector, |ψ⟩, representing the entire cellular population in a high-dimensional Hilbert space.

For this controlled benchmark, we defined cell type 1 (CT1) using a 2-gene feature set (*G*_CT1_ = {*g*_50_, *g*_90_}) and cell type 2 (CT2) using a 5-gene feature set (*G*_CT2_ = {*g*_60_, *g*_70_, *g*_71_, *g*_80_}). This configuration resulted in a 7-qubit system, where the first two qubits represent CT1 and the remaining five represent CT2.

**Fig. 2:**
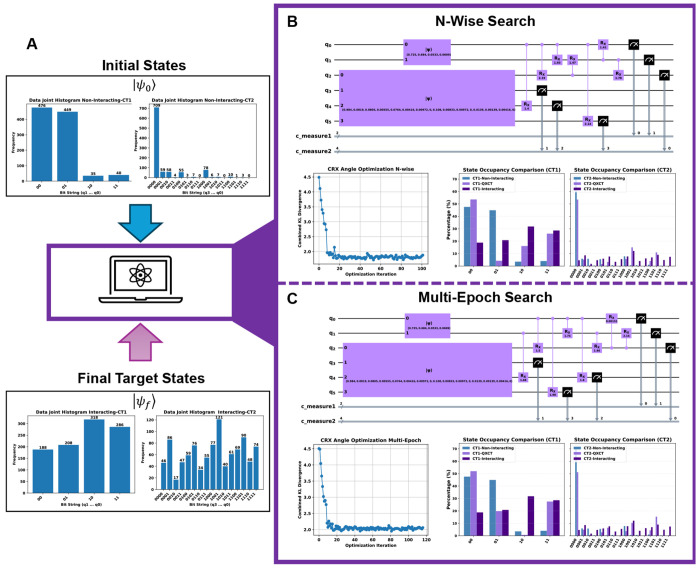
Validation of QuantumXCT on synthetic single-cell data. Our framework successfully learns the transformation from a non-interacting to an interacting cellular state using simulated data with ground-truth rules. **(A) Optimization Workflow.** Input joint histograms of non-interacting cells define the **Initial States** (|Ψ0⟩), while interacting cell histograms define the **Final Target States** (|Ψf⟩). The objective is to identify a unitary transformation, *U* (*τ*_opt_, *θ*_opt_), that evolves the initial distribution to best match the target. **(B) N-Wise Search Performance.** Results for the Iterative Local Search (Algorithm 1, *n* = 2). The panel shows the discovered quantum circuit topology, the KL divergence convergence during angle optimization, and the final state occupancy. The optimized QXCT distribution (light purple) shows high fidelity to the “Interacting” ground-truth (dark purple). **(C) Multi-Epoch Search Performance**. Corresponding results for the Multi-Epoch Search (Algorithm 2). Despite identifying a topologically distinct entangling circuit, the model achieves comparable convergence and occupancy matching. Both heuristics validate the framework’s ability to learn the underlying dynamics and identify key regulatory nodes.

As illustrated in Figure 2, the joint histograms of non-interacting cells (“Initial States”) were used to construct the initial state vector, $\left|\Psi_{0}\right\rangle$. The histograms from the interacting cells (“Final Target States”) defined the target distributions for the KL divergence cost function. We deployed two of our primary classical search heuristics to find the optimal entangling topology: the N-Wise Search (Algorithm 1, with *n* = 2) and the Multi-Epoch Search (Algorithm 2). Both algorithms were used to discover the optimal unitary transformation, $U\left(\tau_{\text{opt}},\theta_{\text{opt}}\right)$, that evolves the initial state distribution to best match the target distribution, thereby learning the underlying communication rules from the data.

As illustrated in Figure 2A, the joint histograms of non-interacting cells (“Initial States”) were used to construct the initial state vector, |Ψ_0_⟩, while the histograms from interacting cells (“Final Target States”) defined the target distributions for the KL divergence cost function. We deployed two of our primary classical search heuristics—the N-Wise Search (Algorithm 1, with *n* = 2) and the Multi-Epoch Search (Algorithm 2)—to discover the optimal entangling topology. Both algorithms were used to identify the optimal unitary transformation, $U\left(\tau_{\text{opt}},\theta_{\text{opt}}\right)$, that evolves the initial state distribution to best match the target distribution, thereby learning the underlying communication rules from the data. Crucially, both heuristics successfully converged to high-fidelity solutions, despite discovering distinct circuit topologies (Fig. 2B, C).

#### 3.1.2 Fidelity of Learned Distributions and Topological Interpretability

The optimization results for the synthetic benchmark demonstrate that QuantumXCT successfully learned the underlying causal structure of the cellular system. As shown in Figure 2, both the N-Wise and Multi-Epoch search heuristics converged to low KL divergence minima. While not a perfect replication, the output distributions of the optimized circuits (labeled “QXCT”) correctly modeled the key transformations from the non-interacting baseline. Both algorithms learned to suppress dominant non-interacting states while increasing the populations of interaction-induced states, demonstrating the model’s ability to capture the directional shift in the high-dimensional probability landscape.

To validate the biological relevance of the discovered circuits, we mapped the entangling gates to the gene-qubit assignments: {*q*_0_ : *g*_50_, *q*_1_ : *g*_90_} for CT1, and {*q*_2_ : *g*_60_, *q*_3_ : *g*_70_, *q*_4_ : *g*_71_ , *q*_5_ : *g*_80_} for CT2.

In the **N-Wise Search** (Fig. 2B), the algorithm discovered a set of entanglements that represent a compressed, functionally equivalent model of the ground-truth pathways. It perfectly recovered the primary ligand-receptor interaction by entangling *q*_0_ with *q*_2_ (*g*_50_ → *g*_60_). Instead of learning the entire multi-step cascade literally, the algorithm utilized powerful “functional shortcuts.” For example, it directly entangled the initial driver *q*_0_ (*g*_50_) with the downstream gene *q*_4_ (*g*_71_), effectively summarizing the initial part of the CT2 GRN defined in Appendix F.3. Similarly, it captured the full intercellular signaling arc by linking CT2’s receptor *q*_2_ (*g*_60_) directly to CT1’s final target *q*_1_ (*g*_90_). Most remarkably, the algorithm uncovered the complex feedback logic by entangling both the intercellular source *q*_5_ (*g*_80_) and the intracellular target *q*_1_ (*g*_90_) back to the initial driver *q*_0_ (*g*_50_). This demonstrates that the PQC learns the most efficient quantum operations that enforce the co-dependencies between key regulatory nodes (*g*_50_, *g*_80_, *g*_90_), rather than simply recapitulating a biological wiring diagram.

The **Multi-Epoch Search** (Fig. 2C) arrived at a topologically distinct solution that nonetheless achieved comparable fidelity and reinforced the same biological principles. This heuristic identified *g*_50_ as a central “hub” by entangling its corresponding qubit (*q*_0_) with multiple downstream genes in the CT2 cascade (*q*_2_, *q*_4_, *q*_5_). Crucially, despite the different topology, it converged on the same key functional relationships found by the N-Wise search, such as the direct influence of the CT2 cascade on CT1’s target (*q*_2_ → *q*_1_ and *q*_2_ → *q*_0_). The emergence of these consistent entangling patterns across different search algorithms strongly suggests that our framework is robustly uncovering the dominant correlations dictated by the underlying GRN and CCC channels.

### 3.2 Single-Cell Communication Inference using Ovarian Cancer Cell Lines

To demonstrate utility in a real biological setting, we applied QuantumXCT to an ovarian cancer dataset in which cancer cells interact with fibroblasts, inducing their transition into cancer-associated fibroblasts (CAFs) [26]. In this case study, the mono-cultured state represents cell lines grown separately, thus with no CCC, whereas co-culture represents the interacting state where CCC drives the CAF phenotype. We hypothesize our framework is well-suited to model the shifts between states by learning an optimal entangling topology (U(τ,θ)) that captures the joint effects of intracellular GRNs and intercellular ligand-receptor (LR) interactions.

**Fig. 3:**
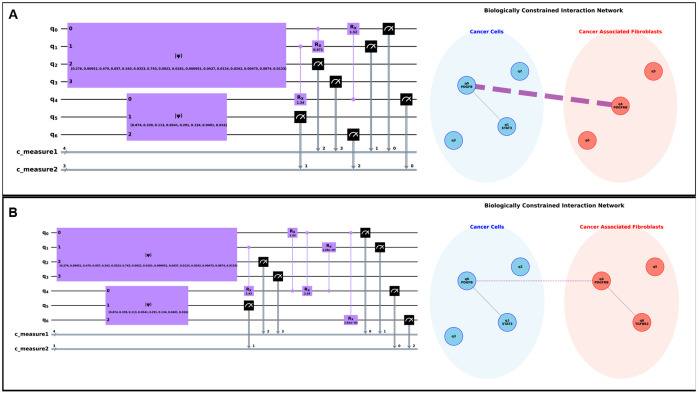
QuantumXCT discovers a core communication hub in an ovarian cancer dataset. Our framework was applied to scRNA-seq data from cancer cells and fibroblasts to model their interaction. **(A) N-Wise Algorithm Results.** The parsimonious 3-gate quantum circuit (left) discovered by the Iterative Local Search and the corresponding biologically constrained interaction network (right). The network plot visualizes the primary intercellular link (PDGFB-PDGFRB) and the intracellular GRN connections within the identified {PDGFB, PDGFRB, STAT3} hub. **(B) Multi-Epoch Algorithm Results.** The larger 5-gate circuit is generated by the stochastic search heuristic. Although topologically different, the resulting network converges on the same core hub, highlighting the robustness of the framework. This solution proposes an additional link to TGFBR2, whose low impact is quantified by the contribution analysis in Table 2, demonstrating the method’s ability to distinguish driver interactions from less significant ones.

For this purpose, we used a moderate density matrix cutoff (*δ_ρ_* > 0.01) to prune the initial search space, focusing the algorithms on the most significant potential interactions. We then applied two primary search heuristics to identify potential CCC interactions; the results are shown in Fig. 3, with a detailed breakdown of gate contributions provided in Table 2.

The **N-Wise Search** (Fig. 3A) converged on a highly parsimonious 3-gate topology that reduced the KL divergence from an initial 0.317 to an optimal 0.093. The resulting network correctly identified a core communication hub consisting of PDGFB, PDGFRB, and STAT3. The ablation analysis (Table 2) revealed that two interactions, PDGFRB → PDGFB and STAT3 → PDGFRB, were responsible for over 90% of the total cost function reduction, highlighting them as the dominant regulatory axes in this model. The biologically constrained network plot visualizes this, showing the known intercellular PDGFB-PDGFRB link as the primary communication channel.

The **Multi-Epoch Search** (Fig. 3B) discovered a larger, 5-gate topology, and reinforced the same core biological finding. This algorithm also identified the PDGFB-PDGFRB-STAT3 hub as the essential component, with the same two interactions accounting for the vast majority of the KL reduction (from 0.326 to 0.079). Notably, this more exploratory search also proposed links such as PDGFB → TGFBR2.

A key insight comes from our two-stage optimization strategy. The initial topology search was performed with fixed CRX(*π*/2) gates to efficiently find a promising interaction structure. The final ablation analysis (Table 2), however, was performed after a fine-tuning step where all gate angles were optimized together, starting from zero. This allowed us to dissect the true contribution of each interaction. The Multi-Epoch strategy revealed that while the PDGFB → TGFBR2 link may have been beneficial during the initial search, its final optimized contribution was negligible and slightly increased the KL divergence (+0.014). This demonstrates the power of our framework to not only discover potential topologies but also to post-analytically distinguish core, important driver interactions from those that are redundant or non-essential in the final optimized model.

Ultimately, both the distinct algorithms converged on the same core biological insight: the cancer → CAF polarization was driven by a CCC feedback loop involving PDGFB, PDGFRB, and STAT3. This confirms the robustness of the QuantumXCT framework and its ability to distill complex quantum entanglements into interpretable biological networks.

## 4 Discussion

The emergence of single-cell transcriptomics has necessitated a shift from descriptive biology toward predictive, dynamical modeling. However, the prevailing reliance on static LR databases inherently limits the discovery of *de novo* or context-specific cellular communication channels [1, 6]. Here, we introduce QuantumXCT, a hybrid quantum-classical generative framework that reframes CCC not as a search for interacting pairs, but as a learning unitary transformation between cellular probability distributions.

### 4.1 Interpretability of Quantum-to-Biological Transcription

A recurring critique of QML is the “black-box” nature of parameterized circuits [8, 9]. We address this directly by demonstrating that the optimized entangling topology ( $\tau_{\text{opt}}$ ) is not merely a computational artifact, but a structured representation of biological logic. By mapping the discovered gates back onto a biologically constrained interaction network (Fig. 3), we obtain a human-interpretable “relay map” of intercellular interaction.

Importantly, our sequential gate contribution analysis (Table 2) allows us to “ablate” the model post-optimization. In the ovarian cancer model, the vast ma jority of the KL divergence reduction, nearly 90%, was driven by the specific PDGFB-PDGFRB-STAT3 hub [26]. This indicates that our quantitative ranking separate high-impact “driver” interactions from secondary “passenger” correlations that can confound classical co-expression benchmarks [4, 6]. Whereas classical methods such as CellChat [1, 2] report a communication probability based on the co-expression of known LR pairs, QuantumXCT quantifies the *functional contribution* of each interaction to the global transcriptomic state change, as evidenced by the sequential ablation results in Table 2. This shift from co-expression to functional impact constitutes the central methodological advance of the framework. Note that, in this work, “impact” refers to changes in cellular state distributions rather than experimentally validated functional or phenotypic effects.

### 4.2 Algorithmic Robustness and Convergence

The stability of QuantumXCT is reflected in the convergence of disparate search heuristics on the same biological ground truth [27]. Although the N-Wise (Algorithm 1) and Multi-Epoch (Algorithm 2) searches produced topologically distinct circuits, both yield functionally equivalent models of the PDGFB signaling axis. This suggests that the quantum state space is naturally conducive to representing the underlying network connectivity, such that multiple entangling paths can successfully encode the same high-dimensional distributional shift [8].

### 4.3 Binarization as a Biologically Motivated Encoding

A natural question concerns whether the binarization step—converting log-normalized expression values to an active/inactive binary state—discards biologically meaningful gradients of expression. We argue that for the specific purpose of quantum state encoding this compression is not only tractable but biologically principled. Single-cell transcriptomic data are inherently sparse, and zero observations following log1p-normalization reflect genuine low-expression states rather than purely technical dropout [11]. Binary representations of scRNA-seq data faithfully capture cell-type-level biological variation [12], and as scRNA-seq datasets grow larger and sparser the downstream conclusions drawn from binarized representations remain consistent with count-based analyses [28]. The active/inactive threshold moreover mirrors the characteristic ON/OFF expression switching observed in regulatory genes at single-cell resolution [13]. Importantly, the QuantumXCT framework does not require binarization to recover quantitative gradients of interaction strength. This is instead captured through the continuous optimization of gate angles *θ* in Section 2.3. The binarization step defines the *topology* of the probability landscape, e.g., which cellular states are accessible and with what frequency, while the continuous parameters learn the *strength* of the transitions between those states. Future extensions could explore multi-level quantization of expression (e.g., low/medium/high) at the cost of exponentially larger qubit registers, requiring advances in quantum hardware beyond the current NISQ era [29].

### 4.4 Marginal Approximation, Scalability, and the Path to Full Joint Inference

The cost function in Eq. 5 minimizes the sum of KL divergences [14] between the *marginal* output distributions of the PQC and the empirical target, evaluated independently for each cell type. This design is not merely a computational convenience but a biological necessity: the two cell populations are experimentally distinct, each with their own gene selection, cell count, and expression landscape. The transcriptomic state of CT1 is encoded in a matrix of dimensions $n_{\text{cells}}^{\text{CT1}}\times g_{\text{CT1}}$, and that of CT2 in a matrix of dimensions $n_{\text{cells}}^{\text{CT2}}\times g_{\text{CT2}}$, where the gene sets and cell counts need not be equal. Because each cell type probability distribution is defined locally—over the activity states of its own gene set and measured only from its own cells—the two marginal distributions are naturally isolated, and each KL divergence term measures a well-defined, self-contained discrepancy. The joint quantum state $\left|\Psi_{\text{Mo}}\right\rangle =\left|\psi_{\text{CT1}}^{\text{Mo}}\right\rangle ⊗\left|\psi_{\text{CT2}}^{\text{Mo}}\right\rangle$ is then constructed via the tensor (Kronecker) product of these two independently defined state vectors (Eq. 4), encoding their non-interacting baseline as a separable state. The unitary U(τ,θ) is then responsible for introducing the entanglement that models cross-cell-type communication, and the optimizer minimizes both marginal KL terms simultaneously, forcing the circuit to learn inter-register dependencies without ever requiring a joint measurement. This architecture is therefore not an approximation of a joint ob jective but the correct formulation for the biological problem: the distributions are genuinely local to their respective cell types, and the entangling topology τ *is* the learned model of the communication between them. The convergence of independently run algorithms to the same PDGFB-PDGFRB-STAT3 hub [26] confirms that simultaneously minimizing both marginal terms is sufficient to identify the dominant functional axes of intercellular signaling. Nonetheless, future work with fault-tolerant quantum hardware could explore joint tomographic approaches to verify higher-order multi-gene dependencies that marginal objectives encode only implicitly [9, 29].

A related scalability boundary was observed in our evaluation of Algorithm 3 (Appendix E). When the initial candidate set from the Δ*ρ* pruning step exceeds approximately 30 gates, the corresponding QUBO matrix [15] becomes intractable for classical post-processing: the number of possible orderings of the selected gates grows factorially with *k*, and gate sets of *k* > 8–10 are not reliably handled by the permutation stage [18, 19]. In practice on the ovarian cancer dataset, Algorithm 3 reduced a candidate pool to 6 gates, from which the quantum solver returned a 5-gate solution—yielding no meaningful parsimony gain over Algorithm 2 [17], which found a comparable topology without the VQE/QAOA overhead. In the current NISQ era [29], this tradeoff is unfavorable for small gene sets. The QUBO formulation becomes genuinely advantageous, however, when the pre-selection step can provide thousands of candidate interactions—a scenario that becomes realistic on fault-tolerant devices capable of screening large interaction spaces in superposition. We therefore regard Algorithm 3 as a forward-looking component: not yet competitive at current hardware scales, but a natural target for quantum advantage as devices mature beyond the NISQ era [9].

### 4.5 Limitations and the “Qubit Bottleneck”

Despite its predictive power, QuantumXCT is currently bounded by the “qubit bottleneck” inherent in the NISQ era [29]. The exponential scaling of the Hilbert space restricts our current implementation to targeted gene sets—typically *N* + *M* ≤ 10 qubits, balanced across the two cell types. While this requires a focused pre-selection of genes, it also serves as a form of biological feature compression: operating in a low-qubit, high-information regime forces the model to identify the most parsimonious regulatory bottlenecks and helps mitigate the over-parameterization common in classical deep learning models [9].

Exploratory analyses applying CellChat [1, 2] and scTenifoldXct [5] to the same ovarian cancer dataset are provided in the accompanying GitHub repository. Both methods recover the PDGFB–PDGFRB interaction, but they do so by testing co-expression of known pairs rather than learning the distributional shift between cellular states. CellChat assigns this interaction a low communication probability, consistent with its mass-action co-expression scoring [1], whereas scTenifoldXct detects it through manifold alignment of GRNs [5]. QuantumXCT addresses a categorically different question: not *which pairs are co-expressed, but how much each interaction contributes to the global transformation of the transcriptomic state*. The sequential gate ablation (Table 2) demonstrates that the PDGFB–PDGFRB–STAT3 axis accounts for nearly 90% of the KL divergence reduction—a functional quantification that is structurally inaccessible to co-expression frameworks regardless of their parameterization [6].

## 5. Conclusion

QuantumXCT represents a tectonic shift in how cellular behavior is inferred from transcriptomics. Unlike traditional approaches that merely “find” interactions by matching ligands and receptors in curated databases [1, 6], our framework models how cellular state distributions change between non-interacting and interacting conditions, and *learns* the system-level impact of an interaction on the cell’s full high-dimensional state. By treating CCC as a generative, probabilistic transformation, we move beyond static database matching toward a truly data-driven discovery platform. Our results on both synthetic and real datasets demonstrate that quantum circuits can capture the directional causality of signaling feedback loops, consistent with results from our companion quantum generative simulation study [7]. As quantum hardware continues to scale beyond the NISQ era [29], we anticipate that this framework will become a cornerstone for uncovering the latent “rules” governing complex cellular systems in oncology, immunology, and regenerative medicine.

## Figures and Tables

**Table 1:** Comparison of Topology Search Algorithms

Algorithm	Strengths	Weaknesses
**1. Iterative Local Search**	• Holistically explores the combined selection and permutation space. • Effective at finding complex, order-dependent interactions. • Flexible search through addition, insertion, and deletion moves.	• Computationally expensive, scales with sequence length. • Greedy nature; not guaranteed to find the global optimum.
**2. Multi-Epoch Sequential Construction**	• Computationally faster than full local search. • Stochastic starting points reduce risk of getting stuck in poor local minima. • Good balance of exploration and speed.	• Forward construction is less thorough in exploring permutations. • May miss solutions where a key gate must be placed early in the sequence.
**3. QUBO-based Variational Selection**	• Maps the hard gate-selection problem to a physical ground state problem (path to quantum advantage). • Can efficiently search over a vast number of gate combinations. • Can be combined with classical methods for a hybrid approach.	• Two-stage process; decouples selection from ordering. • Classical permutation post-processing is intractable for large numbers of selected gates (*k* > 8–10). • VQE/QAOA are heuristic and not guaranteed to find the true ground state.

**Table 2:** Sequential Gate Contribution Analysis for Final Optimized Topologies. This ablation study reveals the true contribution of each interaction after fine-tuning, allowing for the identification of the most impactful gates based on their percent contribution to the total KL reduction.

Algorithm	Source	Target	*θ_i_* (rad)	KL Value	KL Δ	% Contrib.
***N-Wise Search***						
	Baseline	—	—	0.317	0.000	0.0%
	STAT3	PDGFRB	1.345	0.255	−0.062	19.4%
	PDGFB	STAT3	0.971	0.241	−0.015	4.6%
	PDGFRB	PDGFB	1.523	0.093	−0.147	46.5%

***Multi-Epoch Search***						
	Baseline	—	—	0.326	0.000	0.0%
	STAT3	PDGFRB	1.416	0.255	−0.071	21.8%
	PDGFRB	PDGFB	1.433	0.082	−0.173	53.0%
	PDGFB	PDGFRB	2.260	0.086	+0.004	1.2%
	PDGFRB	STAT3	1.3e-07	0.079	−0.007	2.0%
	PDGFB	TGFBR2	1.8e-05	0.093	+0.014	4.3%

## Data Availability

Our algorithm is publicly available on GitHub https://github.com/cailab-tamu/QuantumXCT along with the simulated data used in the study. The ovarian cancer scRNA-seq dataset used in this study is available at GSE224333.

## Appendix A Common Algorithmic Components

All algorithms developed for discovering the optimal entangling topology, *τ*, share foundational components for circuit construction and evaluation. This section details these shared modules.

### A.1 Quantum Circuit Construction and Evaluation

The evaluation of any candidate topology, *τ*, involves constructing the corresponding quantum circuit, simulating it, and calculating a cost.

#### A.1.1 Circuit Construction

A trial quantum circuit, U(τ), is assembled for a given sequence of entangling gates, $\tau =\left(G_{k},\ldots ,G_{1}\right)$. The unitary operator is constructed as $(\tau )=U_{G_{k}}\circ \cdots \circ U_{G_{1}}$. During the discrete topology search phase, each gate Gi in the sequence is implemented as a Controlled-RX gate with a fixed angle, CRX(*π*/2). This strategic choice was made because a standard CNOT gate (equivalent to CRX(*π*)) creates maximal entanglement, which might be too “coarse” a tool for this problem. We reasoned that a discrete search using CNOTs might overlook weaker, yet biologically significant, correlations. By using CRX(*π*/2), we allow the topology search to identify potential interactions based on their ability to create partial or “mixed” entanglement, thereby capturing a wider range of potential inter- and intra-state interactions that minimize the cost function.

The final state of the system before measurement is $\left|\psi^{\prime }\right\rangle =U(\tau )\left|\Psi_{\text{Mo}}\right\rangle$. Measurement operations are appended to each logical register (CT1 and CT2).

#### A.1.2 Cost Function Evaluation

The fitness of a circuit is quantified by its cost, ℒ(τ).

1. **Quantum Simulation:** The circuit U(τ) is executed for nshots, yielding measurement counts $C_{sim}^{{\mathrm{CT}}_{k}}(s)$ for each state *s* in each logical register *k*.
2. **Quantum Inferred Distribution Sampling:** The counts are normalized to produce marginal probability distributions from quantum sampled histograms (related to the amplitudes Eqs. 1 and 2): $P_{\psi^{\prime }}^{{\mathrm{CT}}_{k}}(s)=C_{\text{sim}}^{{\mathrm{CT}}_{k}}(s)/$ nshots.
3. **KL Divergence Calculation:** The total cost is the sum of KL divergences between the simulated and target distributions (from Eq. 5):
  (A1) $$D_{\mathrm{KL}}\left(P_{\psi^{\prime }}^{{\mathrm{CT}}_{k}}\|Q_{\mathrm{Co}}^{{\mathrm{CT}}_{k}}\right)=\sum_{s}P_{\psi^{\prime }}^{{\mathrm{CT}}_{k}}(s)log\left(\frac{P_{\psi^{\prime }}^{{\mathrm{CT}}_{k}}(s)}{Q_{\mathrm{Co}}^{{\mathrm{CT}}_{k}}(s)}\right)$$

## Appendix B Heuristic Pruning of the Gate Search Space

All topology search algorithms described in this work operate not on the complete set of all possible entangling gates, but on a reduced, high-potential candidate set, 𝒞. This initial pruning step is critical for making the subsequent combinatorial and permutational searches tractable.

We guide the search by analyzing the difference between the global density matrices of the non-interacting and interacting systems. First, we define the global state vectors for the mono-culture (initial) and co-culture (target) systems as the tensor products of their respective individual state vectors, which are derived from the experimental data as described in the main text (Eqs. 1 and 2):

(B2) $$\left|\Psi_{\mathrm{Mo}}\right\rangle =\left|\psi_{\mathrm{CT}1}^{\mathrm{Mo}}\right\rangle ⊗\left|\psi_{\mathrm{CT}2}^{\mathrm{Mo}}\right\rangle$$

(B3) $$\left|\Psi_{\mathrm{Co}}\right\rangle =\left|\psi_{\mathrm{CT}1}^{\mathrm{Co}}\right\rangle ⊗\left|\psi_{\mathrm{CT}2}^{\mathrm{Co}}\right\rangle$$

The corresponding density matrices are then defined by the outer product, ρ=|Ψ⟩⟨Ψ|. We compute the difference between the target and initial density matrices:

(B4) $$\Delta \rho =\rho_{\mathrm{Co}}-\rho_{\mathrm{Mo}}$$

The matrix Δ*ρ* highlights the specific state-to-state transitions (i.e., the off-diagonal elements) and changes in state populations (i.e., the diagonal elements) required to evolve the system from the non-interacting to the interacting state. By applying a threshold to the elements of Δ*ρ*, we identify the most significant state-to-state transitions. From this, we generate a reduced candidate set, C , where each element is a (control, target) qubit pair that specifies a potential CRX(*π*/2) gate. These pairs are selected because they are the most likely to facilitate the identified transitions. This pruned set 𝒞 serves as the direct input for all subsequent topology search algorithms.

## Appendix C Algorithm 1: Iterative Local Search

This appendix provides a detailed description of the *Iterative Local Search* algorithm, a classical heuristic for topology discovery. The algorithm begins with the pruned candidate set 𝒞 (Appendix B) and uses the evaluation pipeline from Appendix A. It directly addresses the combined gate selection and ordering problem by iteratively and sequentially applying three types of moves—insertion, addition, and deletion—to refine a sequence of entangling gates.

### C.1 Algorithmic Workflow: Sequential Local Search

The algorithm operates within a main iterative loop that continues as long as any move within an iteration successfully improves the cost function. Unlike a standard local search that evaluates all neighbors before making a move, this algorithm executes three distinct phases in a fixed sequence within each iteration, immediately updating the working solution if a phase is successful. This aggressive, sequential approach is designed to rapidly descend the cost landscape, as outlined in Algorithm 1.

**Algorithm 1:** Iterative Local Search for Topology Discovery

	**Input:** Candidate gate set 𝒞, Target distributions *Q*_Co_, Permutation length *n*_choose_, Significance threshold kl_tol.	
	**Output:** Optimal sequence *τ*_best_, Final cost *L*_best_.	
1:	**Initialize:** $\tau_{\text{best}}\leftarrow \emptyset ,L_{\text{best}}\leftarrow ℒ(\emptyset )$, improvement_made ← True.	
2:	**while** improvement_found **do**	
3:	improvement_made ← False	
4:	▷ *Phase 1: Single Gate Insertion*	
5:	(*τ*_cand_, *L*_cand_) ← FindBestSingleInsertion(*τ*_best_, 𝒞, kl_tol)	
6:	**if** *L*_cand_ < (*L*_best_ – kl_tol) **then**	
7:	*τ*_best_ ← *τ*_cand_, *L*_best_ ← *L*_cand_, improvement_made ← True	
8:	**end if**	
9:	▷ *Phase 2: N-wise Permutation Addition (on updated sequence)*	
10:	(*τ*_cand_, *L*_cand_) ← FindBestPermutationAddition(*τ*_best_, 𝒞, *n*_choose_, kl_tol)	
11:	**if** *L*_cand_ < (*L*_best_ – kl_tol) **then**	
12:	*τ*_best_ ← *τ*_cand_, *L*_best_ ← *L*_cand_, improvement_made ← True	
13:	**end if**	
14:	▷ *Phase 3: Single Gate Deletion (on updated sequence)*	
15:	(*τ*_cand_, *L*_cand_) ← FindBestSingleDeletion(*τ*_best_)	
16:	**if** *L*_cand_ < (*L*_best_ – *ϵ*_prune_) **then**	▷ *ϵ*_prune_ is a small positive constant
17:	*τ*_best_ ← *τ*_cand_, *L*_best_ ← *L*_cand_, improvement_made ← True	
18:	**end if**	
19:	**end while**	
20:	**Return** *τ*_best_, *L*_best_	

### C.2 Phase Descriptions

Each phase of the iteration attempts to find the best possible move of a specific type and accepts it based on a defined criterion.

#### C.2.1 Phase 1: Single Gate Insertion

This move probes the importance of gate ordering by finding the best single gate to insert and its optimal position within the current sequence, $\tau_{\text{current}}$. The search for the optimal gate-index pair (*G**, *i**) is defined as:

(C5) $$\left(G^{*},i^{*}\right)=\arg\underset{G\in 𝒞\setminus \tau_{\text{current}},i\in \left[0,\left|\tau_{\text{current}}\right|\right]}{\min}ℒ\left(\mathrm{Insert}\left(G,\tau_{\text{current}},i\right)\right)$$

The resulting sequence $\tau_{\text{insert}}=Insert\left(G^{*},\tau_{\text{current}},i^{*}\right)$ is immediately adopted if it provides a cost reduction greater than the significance threshold, i.e., if $ℒ\left(\tau_{\text{insert}}\right)<$ ( $ℒ\left(\tau_{\text{current}}\right)-kl\_\mathrm{tol})$.

#### C.2.2 Phase 2: N-wise Permutation Addition

Operating on the potentially updated sequence from the previous phase, this move considers adding a short, ordered sequence of new gates. The length of this sequence, *n*, is a user-definable parameter (n_choose). This phase searches over all permutations *p* of length *n* drawn from the remaining candidate gates ${𝒞}_{\text{rem}}=𝒞\setminus \tau_{\text{current}}$ The best permutation *p** to append is found by solving:

(C6) $$p^{*}=\arg\;\underset{p\in {𝒫}_{n}\left({𝒞}_{\text{rem}}\right)}{\min}ℒ\left(\tau_{\text{current}}⊕p\right)$$

where ⊕ denotes sequence concatenation. The resulting sequence $\tau_{\text{add}}=\tau_{\text{current}}⊕p^{*}$ is immediately adopted if it meets the significance threshold.

This move explicitly tests for synergistic effects between small, ordered groups of new gates. While the algorithm supports any integer *n*, the computational cost of this phase scales factorially with *n* and polynomially with the size of ${𝒞}_{\mathrm{rem}}$. Therefore, for practical applications, small values of *n* are necessary. In this work, we set *n* = 2, focusing the search on pairwise interactions, which we found provides a good balance between uncovering synergistic effects and maintaining computational tractability.

#### C.2.3 Phase 3: Single Gate Deletion

Finally, this move provides a crucial pruning mechanism to correct for earlier greedy steps. It finds the optimal index *i** of a gate to remove from the current sequence by finding the move that yields the absolute lowest cost:

(C7) $$i^{*}=arg\underset{i\in \left[0,\left|\tau_{\text{current}}\right|-1\right]}{\min}ℒ\left(\mathrm{Delete}\left(\tau_{\text{current}},i\right)\right)$$

The resulting sequence, $\tau_{\text{del}}=Delete\left(\tau_{\text{current}},i^{*}\right)$, is adopted if its cost is lower than the current cost by more than a small, fixed margin $\left(ℒ\left(\tau_{\text{del}}\right)<ℒ\left(\tau_{\text{current}}\right)-\epsilon_{\text{prune}}\right)$. This less strict criterion allows the algorithm to remove redundant gates to favor parsimony, even for marginal cost improvements.

## Appendix D Algorithm 2: Multi-Epoch Sequential Construction

This appendix provides a detailed description of the *Multi-Epoch Sequential Construction* algorithm. This heuristic is designed as a faster, stochastic alternative to the exhaustive local search, aiming to balance a broad exploration of the solution space with a final selection principle that favors model parsimony. The algorithm begins with the pruned candidate set 𝒞 (Appendix B) and utilizes the evaluation pipeline from Appendix A.

### D.1 Algorithmic Workflow

The algorithm is composed of three interconnected phases. First, it explores the solution space via multiple **Forward Greedy Construction** epochs; an epoch only proceeds if the initial random gate improves upon the baseline. Second, an **Immediate Backward Greedy Refinement** is triggered *if* a forward search yields a new best-so-far solution; this phase removes irrelevant gates to ensure each addition in the sequence is strictly necessary. Finally, after all epochs are complete, a global selection phase based on Occam’s Razor determines the final result. The process is outlined in Algorithm 2.

**Algorithm 2:** Multi-Epoch Construction with Path Refinement and Occam’s Razor

	**Input:** Candidate gate set 𝒞, Target distributions *Q*, Epochs *N*_epochs_, Threshold *ϵ* (kl_tol).	
	**Output:** Optimal gate sequence *τ*^∗^, Final cost *L**.	
1:	**Initialize:** $\tau_{\text{best}}\leftarrow \emptyset ,L_{\text{best}}\leftarrow ℒ(\emptyset ),{ℋ}_{\text{eval}}\leftarrow \left\{\left(L_{\text{best}},\emptyset \right)\right\},L_{\emptyset }\leftarrow L_{\text{best}}$	
2:	${𝒞}_{\mathrm{shuffled}}$ ← Shuffle(𝒞)	
3:	▷ *Phase 1: Multi-Epoch Exploration & Refinement*	
4:	**for** epoch from 1 to min(*N*_epochs_, |𝒞|) **do**	
5:	*G*_start_ ← *C*_shuffled_ [epoch]	
6:	*L*_start_ ← ℒ((*G*_start_))	
7:	**if** *L*_start_ < L_∅_ **then**	▷ Proceed only if *G*_start_ improves on baseline
8:	▷ Step 1.1: Greedy Forward Construction	
9:	$\left({ℋ}_{\text{path}},\tau_{\text{path}},L_{\text{path}}\right)\leftarrow \mathrm{GreedyForward}\left(\left(G_{\text{start}}\right),𝒞\right)$	
10:	${ℋ}_{\mathrm{eval}}\leftarrow {ℋ}_{\mathrm{eval}}\cup {ℋ}_{\mathrm{path}}$	
11:	**if** *L*_path_ < *L*_best_ **then**	▷ New global champion found on this path
12:	*L*_best_ ← *L*_path_, *τ*_best_ ← *τ*_path_	
13:	▷ Step 1.2: Immediate Greedy Removal (Refinement)	
14:	$\left({ℋ}_{\text{rem}},\tau_{\text{rem}},L_{\text{rem}}\right)\leftarrow GreedyRemoval\left(\tau_{\text{best}},L_{\text{best}},\epsilon \right)$	
15:	${ℋ}_{\mathrm{eval}}\leftarrow {ℋ}_{\mathrm{eval}}\cup {ℋ}_{\mathrm{rem}}$	
16:	**if** *L*_rem_ < *L*_best_ **then**	
17:	*L*_best_ ← *L*_rem_, *τ*_best_ ← *τ*_rem_	
18:	**end if**	
19:	**end if**	
20:	**end if**	
21:	**end for**	
22:	▷ *Phase 2: Global Selection via Occam’s Razor*	
23:	Sort ${ℋ}_{\mathrm{eval}}$ by sequence length |*τ*| in ascending order.	
24:	(*τ**, *L**) ← First element of sorted ${ℋ}_{\mathrm{eval}}$	
25:	**for** each (*L*_i_, *τ*_i_) ∈ sorted ${ℋ}_{\mathrm{eval}}$ **do**	
26:	**if** *L_i_* < (*L** – *ϵ*) **then**	▷ Accept longer sequence only if gain > ϵ
27:	*τ** ← *τ_i_*, *L** ← *L_i_*	
28:	**end if**	
29:	**end for**	
30:	**Return:** *τ**, *L**	

### D.2 Phase Descriptions

#### D.2.1 Phase 1: Multi-Epoch Forward Construction and Refinement

This phase explores distinct regions of the solution space by initiating multiple independent, stochastic greedy searches. For each epoch, a candidate gate *G_start_* is selected from the shuffled set ${𝒞}_{\mathrm{shuffled}}$. To maintain computational efficiency, a full forward greedy search is only initiated if this initial single-gate sequence improves upon the baseline empty circuit:

(D8) $$ℒ\left(\left(G_{\text{start}}\right)\right)<ℒ(\emptyset )$$

- **Forward Greedy Construction:** If the starting gate is promising, the algorithm performs an iterative constructive search. At each step *k*, it identifies the optimal gate to append from the candidate set:
  (D9) $$G_{k+1}^{*}=\arg\;\underset{G\in 𝒞\setminus \tau_{k}}{\min}ℒ\left(\tau_{k}⊕G\right)$$
  The new sequence $\tau_{k+1}=\tau_{k}⊕G_{k+1}^{*}$ is accepted if $ℒ\left(\tau_{k+1}\right)<ℒ\left(\tau_{k}\right)$. This process continues until no single gate addition can improve the cost or the maximum depth is reached. The entire history of evaluated sequences is recorded in ${ℋ}_{\mathrm{eval}}$.
- **Immediate Backward Refinement:** If the forward search yields a sequence $\tau_{\text{path}}$ that is a new global champion $\left(ℒ\left(\tau_{\text{path}}\right)<L_{\text{best}}\right)$, a refinement step is triggered to prune redundant gates. Starting with $\tau_{0}=\tau_{\text{path}}$, the algorithm evaluates the removal of every gate in the current sequence to identify the position *i** that yields the minimum cost:
  (D10) $$i^{*}=\arg\;\underset{i\in \left[0,\left|\tau_{k}\right|-1\right]}{\min}ℒ\left(\mathrm{Delete}\left(\tau_{k},i\right)\right)$$
  The pruned sequence $\tau_{k+1}=Delete\left(\tau_{k},i^{*}\right)$ is accepted if and only if $ℒ\left(\tau_{k+1}\right)<ℒ\left(\tau_{k}\right)-\delta$, where *δ* = 0.3 · *ϵ* represents a significance margin. This greedy positional removal continues iteratively until no single gate deletion satisfies the improvement criterion or the minimum depth is reached. This refinement phase ensures that any redundant gates introduced during the constructive phase are eliminated.

#### D.2.2 Phase 2: Final Selection (Occam’s Razor)

To select the final topology *τ**, the algorithm applies a model parsimony principle. The complete history of all unique sequences ${ℋ}_{\mathrm{eval}}$ evaluated during all forward and backward phases is collected and sorted by sequence length |τ| in ascending order.

The algorithm iterates through this sorted list, starting with the simplest model (the empty circuit). A more complex (longer) sequence $\tau_{i}$ is only accepted as the new best solution if its cost provides an improvement greater than the significance threshold *ϵ* (kl_tol):

(D11) $$ℒ\left(\tau_{i}\right)<ℒ\left(\tau^{*}\right)-\epsilon$$

This ensures that the final selected topology *τ** is the most parsimonious model that achieves a significant reduction in cost, effectively preventing over-parameterization and ensuring model robustness.

## Appendix E Algorithm 3: QUBO-based Variational Selection

This appendix details our quantum-native approach, which begins with the pruned candidate set 𝒞 (Appendix B). This strategy reframes the gate selection problem as a Quadratic Unconstrained Binary Optimization (QUBO) problem, decoupling it into a quantum-assisted gate selection and a final classical ordering stage.

### E.1 Stage 1: KL-QUBO Formulation for Gate Selection

The goal of this stage is to map the circuit optimization problem onto a QUBO framework. We define a binary vector x ∈ {0, 1}^N^, where *x_i_* = 1 if the candidate CNOT gate *G_i_* ∈ *C* is included in the circuit for a qubit pair, and *x_i_* = 0 otherwise. The ob jective is to minimize the energy function:

(E12) $$E(x)=\sum_{i=1}^{N}Q_{\mathrm{ii}}x_{i}+\sum_{i<j}Q_{\mathrm{ij}}x_{i}x_{j}$$

The construction of the QUBO matrix Q proceeds in two steps:

#### E.1.1 KL Divergence Matrix Construction

We pre-compute a KL divergence matrix $M_{KL}\in R^{N\times N}$ by evaluating the cost of single gates and gate pairs against the target distributions. Let ℒ(τ) denote the summed KL divergence of a circuit containing the sequence of gates *τ*. The elements of MKL are defined as:

(E13) $${\left(M_{\mathrm{KL}}\right)}_{\mathrm{ij}}=\left\{\begin{array}{ll} ℒ\left(G_{i}\right) & \text{if}\;i=j\\ ℒ\left(G_{i},G_{j}\right) & \text{if}\;i\neq j \end{array}\right.$$

where $L_{\emptyset }=ℒ(\emptyset )$ represents the baseline KL divergence of the circuit with no additional CNOT gates.

#### E.1.2 QUBO Matrix Derivation

The matrix *M_KL_* is transformed into the QUBO matrix *Q* by isolating the individual and synergistic contributions of the gates:

1. **Linear Coefficients (Diagonal Terms):** The diagonal elements *Q_ii_* represent the improvement (or degradation) a single gate provides relative to the baseline:
  (E14) $$Q_{\mathrm{ii}}={\left(M_{\mathrm{KL}}\right)}_{\mathrm{ii}}-L_{\emptyset }$$
2. **Quadratic Coefficients (Off-Diagonal Terms):** For any pair of gates {*G_i_, G_j_*}, the physical circuit must adopt a specific temporal order. To simplify the optimization, we assume the solver utilizes the optimal ordering. We first define the best-order cost:
  (E15) $$L_{\mathrm{best}}(i,j)=\min\left({\left(M_{\mathrm{KL}}\right)}_{\mathrm{ij}},{\left(M_{\mathrm{KL}}\right)}_{\mathrm{ji}}\right)$$
  The interaction term *Q_ij_* is then derived by subtracting the individual costs from the joint cost to isolate the synergy:
  (E16) $$Q_{\mathrm{ij}}=\left(L_{\mathrm{best}}(i,j)-L_{\emptyset }\right)-Q_{\mathrm{ii}}-Q_{\mathrm{jj}}$$

Any sequence that results in an infinite or non-computable KL divergence is assigned a penalty $P\gg {\left|Q_{\mathrm{ij}}\right|}_{\text{max}}$ to ensure the solver avoids invalid gate configurations.

### E.2 Stage 2: Variational Quantum Solution

The QUBO is converted to an Ising Hamiltonian, whose ground state (the optimal gate set) is found using VQE or QAOA.

- **VQE:** We use an EfficientSU2 ansatz with a classical COBYLA optimizer to find the ground state.
- **QAOA:** As a comparison, we use a standard QAOA implementation, also with a COBYLA optimizer.

The output of the quantum solver is a probability distribution over possible solutions. We take the top-k most probable bitstrings as candidate solutions.

### E.3 Stage 3: Hybrid Post-Processing

The quantum solver provides an optimal *set* of gates, but not their order. For each of the top-k gate sets identified, we perform a final classical search to find the optimal permutation. This search is conducted using our **Multi-Epoch Sequential Construction (Algorithm 2)**, which efficiently finds a high-quality ordering for the small, promising set of gates selected by the quantum algorithm. The best sequence found across all top-k VQE solutions is chosen as the final topology.

## Appendix F Spatial Mechanistic Simulation Framework

To benchmark the quantum information-theoretic algorithm’s ability to resolve directional causality, we developed a synthetic tissue framework that explicitly separates spatial signaling from intracellular regulation.

### F.1 Spatial Architecture and Interaction Kernel

The tissue comprises *N* cells *C* with spatial coordinates $P_{i}\in R^{2}$. Intercellular connectivity is governed by a Gaussian decay kernel:

(F17) $$W_{\mathrm{ij}}=\exp\left(-\frac{{\left\|P_{i}-P_{j}\right\|}^{2}}{2\sigma^{2}}\right),\;i\neq j$$

Cells are partitioned into four scenarios: *Interacting Pairs* (A and B clusters within signaling range), *Lone Senders* (*A_lone_*), and *Lone Receivers* (*B*lone**). This setup allows for the evaluation of algorithm robustness against spatial co-occurrence false positives.

### F.2 The Latent Manifold and Causality Chain

The simulation models a continuous latent state $x_{i}\in R^{G}$ representing log-transformed transcriptomic potential. Every gene is initialized at a stochastic baseline ${\overset{‾}{x}}_{g,i}∼𝒩\left(\mu_{0},\sigma_{\text{noise}}\right)$, where *μ_0_* = −2.0 represents “Biological Zero.” Causality advances through a rigid temporal sequence:

1. **Ligand Activation:** Sender cell ligands *x_l,j_* are manually shifted upward from baseline to represent active signal secretion.
2. **Intercellular Signaling** (*S_r,i_*): Receiver cell *i* integrates neighbor signals by bridging the source expression (*x_l,j_*) and induced response (xr,i):
  (F18) $$x_{r,i}={\overset{‾}{x}}_{r,i}+\log\left(1+\alpha \sum_{j\in {𝒞}_{\text{sender}}}W_{\mathrm{ij}}\cdot \exp\left(x_{l,j}\right)\right)$$
3. **Intracellular GRN Propagation:** Downstream targets respond to the receptor via a Gene Regulatory Network (GRN) adjacency matrix *A_gk_*:
  (F19) $$x_{g,i}=x_{g,i}+\sum_{k\in 𝒢}A_{\mathrm{gk}}\cdot \left(x_{k,i}-\mu_{0}\right)\cdot I\left(x_{k,i}>\tau \right)$$
  By subtracting μ0, we ensure the network responds only to relative activation (the “shout”) while ignoring baseline stochasticity (the “hum”).

### F.3 Causal Benchmarking Scenarios

We implement two distinct circuits to challenge the algorithm’s resolution:

1. **Feedback Loop:** A three-stage intercellular circuit:
  (F20) $$\underset{\text{Forward Signaling}}{\underbrace{50_{L}\overset{W_{\mathrm{ij}}}{\to }60_{R}}}⟹\underset{\text{Reactive GRN}}{\underbrace{60\to 70\to \left\{71,72,80_{L}\right\}}}⟹\underset{\text{Retrograde Feedback}}{\underbrace{80_{L}\overset{W_{ji}}{\to }90_{R}}}\text{.}$$
2. **Autonomous Module:** An independent module (73 → {74, 75}) initialized globally to test the decoupling of spatial information from intrinsic mutual information.

### F.4 Measurement Model and Sparsity

To simulate technical capture inefficiency, the latent manifold is projected into observed UMI counts *Y_gi_* via a Negative Binomial distribution with Bernoulli-mask dropout:

(F21) $$Y_{\mathrm{gi}}∼\mathrm{NB}\left(\exp\left(x_{\mathrm{gi}}\right),\phi \right)\cdot \mathrm{Bernoulli}(1-\pi )$$

With *π* = 0.4, the environment features ~ 90% sparsity, forcing the algorithm to distinguish biological signal from technical noise.

**Implementation Note:** The complete Python source code is available at github.com/cailab-tamu/QuantumXCT/python/simCellsSpace.
